# Supplementary material for: Social network analysis for social neuroscientists
Source: Soc Cogn Affect Neurosci. 2020 May 18;16(8):883–901. doi: 10.1093/scan/nsaa069 (PMC8343567; doi:10.1093/scan/nsaa069)
Supplement: nsaa069_Supp [file nsaa069_supp.zip › scan_supplementals_FINAL.pdf]

Supplementary Material

An Example of Other Measures of Centrality: PageRank

As we described in the main text, there are many ways of characterizing centralities (i.e., importances) of people in a social network. We outlined a handful of such measures (specifically, degree, in-degree, out-degree, diffusion centrality, betweenness centrality, and eigenvector centrality) in the main text. We now describe a popular measure of centrality known as PageRank centrality (which is a variant of eigenvector centrality; Gleich, 2015). PageRank centrality (or simply "PageRank") incorporates a probability for the walking robot that we described in the "Centrality" section of the main text to "teleport" to random nodes in a network in addition to traversing the network's edges. A node (e.g., a page on the World Wide Web) tends to be central according to PageRank if it has large in-degree (e.g., many other Web pages point to it) and the incoming edges are from nodes that themselves have a large in-degree (e.g., the Web pages that point to it have a lot of other Web pages that point to them). PageRank takes into consideration both the direction and the weights of edges, and one construes a Web page to be important if many other important Web pages link to it. Suppose that a robot is randomly surfing the Web, so it is randomly walking from one node (i.e., Web page) to another through directed edges (i.e., hyperlinks that point from one Web page to another) and randomly "teleporting" to other Web pages by opening a new browser window. One can calculate the PageRank centrality of a Web page by examining how often the robot visits it, including through teleportation, if it surfs the Web forever (Masuda et al., 2017). PageRank is associated most famously with ranking Web pages, but it has also been applied to investigate questions in a large variety of topics, including ranking the influence of Twitter users, ranking academic journals and doctoral programs, and finding correlated genes and proteins. For a review of PageRank, see Gleich (2015).

Additional Future Directions: Other Methods of Network Analysis

We briefly discuss other methods (including ones that are under rapid development) in network analysis that may be useful for social neuroscientists who are interested in characterizing real-world social networks and relating those characteristics to neuroscientific data. We discuss the potential utility of topological data analysis, community-level characteristics, and analysis of other mesoscale features to study social networks.

Beyond pairwise connectivity in networks. We anticipate that it will be fruitful to examine relationships between nodes beyond the usual pairwise connections. The simplest way to do this is with hypergraphs, which allow edges (which are called “hyperedges” in this context) to connect more than two nodes and are thus useful for representing relationships that involve more than two people (Newman, 2018). One example is a coauthorship network, where a single hyperedge connects all of the coauthors of a manuscript, instead of connecting them through multiple pairwise edges. Another example is a network of college roommates, where it may be desirable to use a single hyperedge to connect all occupants of one room, which may be shared by more than two people. Moreover, it is possible that some pairs of roommates may also be connected directly in a pairwise fashion, so using hypergraphs gives a sensible way to simultaneously include both pairwise connections and other connections in a network structure. A more complicated, but likely very useful, approach to study relationships among arbitrarily many actors in a social network is to use “simplicial complexes” (Ghrist, 2014), an idea from algebraic topology that many researchers have leveraged for “topological data analysis” (Otter et al., 2017; Topaz, 2016). One can use tools from topological data analysis to systematically examine a diversity of structural features of social networks, such as by algorithmically finding topological “holes” (e.g., gaps) in coauthorship networks (Carstens & Horadam, 2013). Perhaps such holes may help uncover barriers to academic collaboration, and it seems plausible to try to relate such topological holes to Burt’s notion of “structural holes” in social networks (Burt, 1992). The most popular approach from topological data analysis is “persistent homology,” which

allows one to track many types of topological holes over multiple scales in a network. We anticipate that persistent homology and other tools from topological data analysis will be used increasingly in the study of social networks. See Topaz (2016) for a brief popular introduction and Otter et al. (2017) for a more mathematical introduction and a tutorial of available software.

Community-level characteristics and mesoscale network structures. As we discussed briefly in the main text, one can examine densely connected communities of nodes in a network. There are numerous algorithms to study community structure; some of them involve assigning nodes to single communities, and others allow overlapping communities (Fortunato & Hric, 2016; Porter et al., 2009). One potential future direction that involves community structure and other large-scale network structures is to simultaneously relate individuals' brain data to features of the local structures of their networks, characteristics of their intermediate-scale (i.e., "mesoscale") structures (such as communities), and global network characteristics of a network. As we discussed in the main text, there exist numerous algorithms for identifying communities in a network (Fortunato & Hric, 2016; Porter et al., 2009). There are also methods for characterizing other types of intermediate-scale structures. One example is "core–periphery structure," in which one attempts to detect one or more cores of densely connected nodes, along with sparsely connected peripheral nodes (Csermely et al., 2013; Rombach et al., 2017). Another example is "role structure," in which one attempts to detect similar role structures of nodes (e.g., perhaps the ego networks of graduate students, postdoctoral scholars, and professors have different structural characteristics), regardless of the density of connections (Rossi & Ahmed, 2015). Future research that integrates tools for detection of communities and other mesoscale structures in networks may be fruitful for elucidating how the features of such large-scale structures impact individuals' cognitive processes and behavior.

References

Burt, R. S. (1992). *Structural Holes: The Social Structure of Competition*. Harvard University Press.

Carstens, C. J., & Horadam, K. J. (2013). Persistent homology of collaboration networks. *Mathematical Problems in Engineering*, *2013*, 815035. https://doi.org/10.1155/2013/815035

Csermely, P., London, A., Wu, L. Y., & Uzzi, B. (2013). Structure and dynamics of core/periphery networks. *Journal of Complex Networks*, *1*(2), 93–123. https://doi.org/10.1093/comnet/cnt016

Fortunato, S., & Hric, D. (2016). Community detection in networks: A user guide. *Physics Reports*, *659*, 1–44. https://doi.org/10.1016/j.physrep.2016.09.002

Ghrist, R. (2014). *Elementary Applied Topology* (edition 1.0). CreateSpace Independent Publishing Platform. https://www.math.upenn.edu/~ghrist/notes.html

Gleich, D. F. (2015). PageRank beyond the Web. *SIAM Review*, *57*(3), 321–363. https://doi.org/10.1137/140976649

Masuda, N., Porter, M. A., & Lambiotte, R. (2017). Random walks and diffusion on networks. *Physics Reports*, *716–717*, 1–58. https://doi.org/10.1016/j.physrep.2017.07.007

Newman, M. E. J. (2018). *Networks* (2nd ed.). Oxford University Press.

Otter, N., Porter, M. A., Tillmann, U., Grindrod, P., & Harrington, H. A. (2017). A roadmap for the computation of persistent homology. European Physical Journal — Data Science, *6*(1), 17. https://doi.org/10.1140/epjds/s13688-017-0109-5

Porter, M. A., Onnela, J.-P., & Mucha, P. J. (2009). Communities in networks. *Notices of the American Mathematical Society*, *56*(9), 1082–1097, 1164–1166. http://www.ams.org/notices/200909/rtx090901082p.pdf

Rombach, P., Porter, M. A., Fowler, J. H., & Mucha, P. J. (2017). Core-periphery structure in networks (revisited). *SIAM Review*, *59*(3), 619–646. https://doi.org/10.1137/120881683

Rossi, R. A., & Ahmed, N. K. (2015). Role discovery in networks. *IEEE Transactions on Knowledge and Data Engineering*, *27*(4), 1112–1131. https://doi.org/10.1109/TKDE.2014.2349913

Topaz, C. (2016). Topological data analysis: One applied mathematician's heartwarming story of struggle, triumph, and ultimately, more struggle. *SIAM Dynamical Systems Web*. https://dsweb.siam.org/The-Magazine/Article/topological-data-analysis
